# Preexisting memory CD4^+^ T cells contribute to the primary response in an HIV-1 vaccine trial

**DOI:** 10.1172/JCI150823

**Published:** 2021-12-01

**Authors:** Suzanne L. Campion, Elena Brenna, Elaine Thomson, Will Fischer, Kristin Ladell, James E. McLaren, David A. Price, Nicole Frahm, Juliana M. McElrath, Kristen W. Cohen, Janine R. Maenza, Stephen R. Walsh, Lindsey R. Baden, Barton F. Haynes, Bette Korber, Persephone Borrow, Andrew J. McMichael

**Affiliations:** 1 Nuffield Department of Clinical Medicine, University of Oxford, Oxford, United Kingdom.; 2 Los Alamos National Laboratory, Santa Fe, New Mexico, USA.; 3 Division of Infection and Immunity and; 4 Systems Immunity Research Institute, Cardiff University School of Medicine, Cardiff, United Kingdom.; 5 Bill & Melinda Gates Medical Research Institute, Cambridge, Massachusetts, USA.; 6 Vaccine and Infectious Disease Division, Fred Hutchinson Cancer Research Center, Seattle, Washington, USA.; 7 Division of Allergy and Infectious Diseases, Department of Medicine, University of Washington, Seattle, Washington, USA.; 8 Department of Medicine, Division of Infectious Diseases, Brigham and Women’s Hospital, Boston, Massachusetts, USA.; 9 Department of Medicine, Duke University School of Medicine, Durham, North Carolina, USA.

**Keywords:** Clinical Trials, Immunology, Cellular immune response, T cells

## Abstract

Naive and memory CD4^+^ T cells reactive with human immunodeficiency virus type 1 (HIV-1) are detectable in unexposed, unimmunized individuals. The contribution of preexisting CD4^+^ T cells to a primary immune response was investigated in 20 HIV-1–seronegative volunteers vaccinated with an HIV-1 envelope (Env) plasmid DNA prime and recombinant modified vaccinia virus Ankara (MVA) boost in the HVTN 106 vaccine trial (clinicaltrials.gov NCT02296541). Prevaccination naive or memory CD4^+^ T cell responses directed against peptide epitopes in Env were identified in 14 individuals. After priming with DNA, 40% (8/20) of the elicited responses matched epitopes detected in the corresponding preimmunization memory repertoires, and clonotypes were shared before and after vaccination in 2 representative volunteers. In contrast, there were no shared epitope specificities between the preimmunization memory compartment and responses detected after boosting with recombinant MVA expressing a heterologous Env. Preexisting memory CD4^+^ T cells therefore shape the early immune response to vaccination with a previously unencountered HIV-1 antigen.

## Graphical abstract

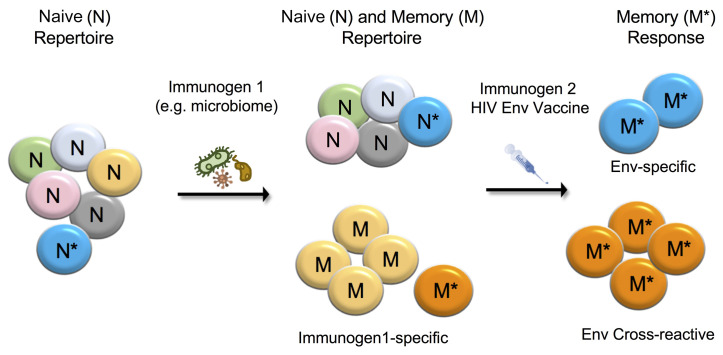

## Introduction

The T cell clonotype repertoire expresses a vast array of receptors that can bind epitopes from almost any foreign immunogen (1). However, immune responses following immunization or infection often focus on relatively few epitopes (2–4). Primary CD4^+^ T cell responses in individuals that share a human leukocyte antigen (HLA) class II allotype generally target common immunodominant epitopes (5). Antigen abundance, processing, presentation, and epitope affinity all influence epitope selection (6). The primary immune response is also influenced by precursor T cell frequency, interclonal competition, and the affinity of the T cell receptor (TCR) for the peptide-HLA complex (2).

Naive and memory T cells can recognize foreign epitopes in humans previously unexposed to the pathogen. Preexisting memory T cells must be primed by cross-reacting antigens (7–10). The number of clonotypes, defined by unique TCRs, is much smaller in any individual than the number of different peptide-HLA complexes that occur in nature but all foreign proteins can stimulate an immune response (11, 12). Immune coverage is therefore facilitated by cross-reactivity, which allows a single TCR to recognize over a million different peptide-HLA complexes (13, 14).

We designed an ancillary study of the human immunodeficiency virus (HIV) Vaccine Trial Network (HVTN) 106 phase I trial to determine if cross-reactive memory CD4^+^ T cells contribute to the primary immune response against newly encountered HIV-1 gp160 envelope (Env). Ultrasensitive quantification and epitope mapping showed that Env-specific naive and memory CD4^+^ T cells were present in unexposed volunteers before immunization. Primary vaccine–elicited immune responses were derived mainly from the preexisting memory pool, as shown by specificity matching and TCR sequencing. These results illustrate original antigenic sin in the context of an early vaccine–induced T cell response (15).

## Results and Discussion

The HVTN 106 vaccine trial and this ancillary study (https://clinicaltrials.gov/ct2/show/NCT02296541) are summarized in Supplemental Table 1; supplemental material available online with this article; https://doi.org/10.1172/JCI150823DS1 Volunteers were randomly assigned to a placebo or 1 of 3 vaccine groups. The vaccine groups received 3 serial immunizations with DNA encoding HIV-1 gp160 Env derived from a B clade transmitted founder virus (NatB), a group M consensus virus (ConS), or a trivalent mosaic sequence designed to optimize global coverage (Mosaic) (16, 17). All 3 groups were then boosted with a recombinant modified vaccinia virus Ankara (MVA) vector expressing *env*/*gag*/*pol* inserts derived from a CRF01 clade AE HIV-1 isolate from Chiang Mai (MVA-CMDR). Placebo controls received saline injections at the same time points. All participants in this study (volunteers, clinical staff, laboratory staff, and authors) were blinded with respect to sample identity until completion of the vaccine trial. Technical limitations precluded the use of peptides spanning all 3 vaccine inserts for the analysis of preexisting responses. Samples were therefore evaluated for CD4^+^ T cell responses to a set of overlapping peptides corresponding to the ConS sequence, which provided the closest match across all 3 vaccine inserts. Later unblinding revealed that 8 volunteers received ConS DNA, 4 NatB DNA, 4 Mosaic DNA, and 4 were placebo controls (Supplemental Table 1).

### Preimmunization T cell responses.

**Figure 1:**
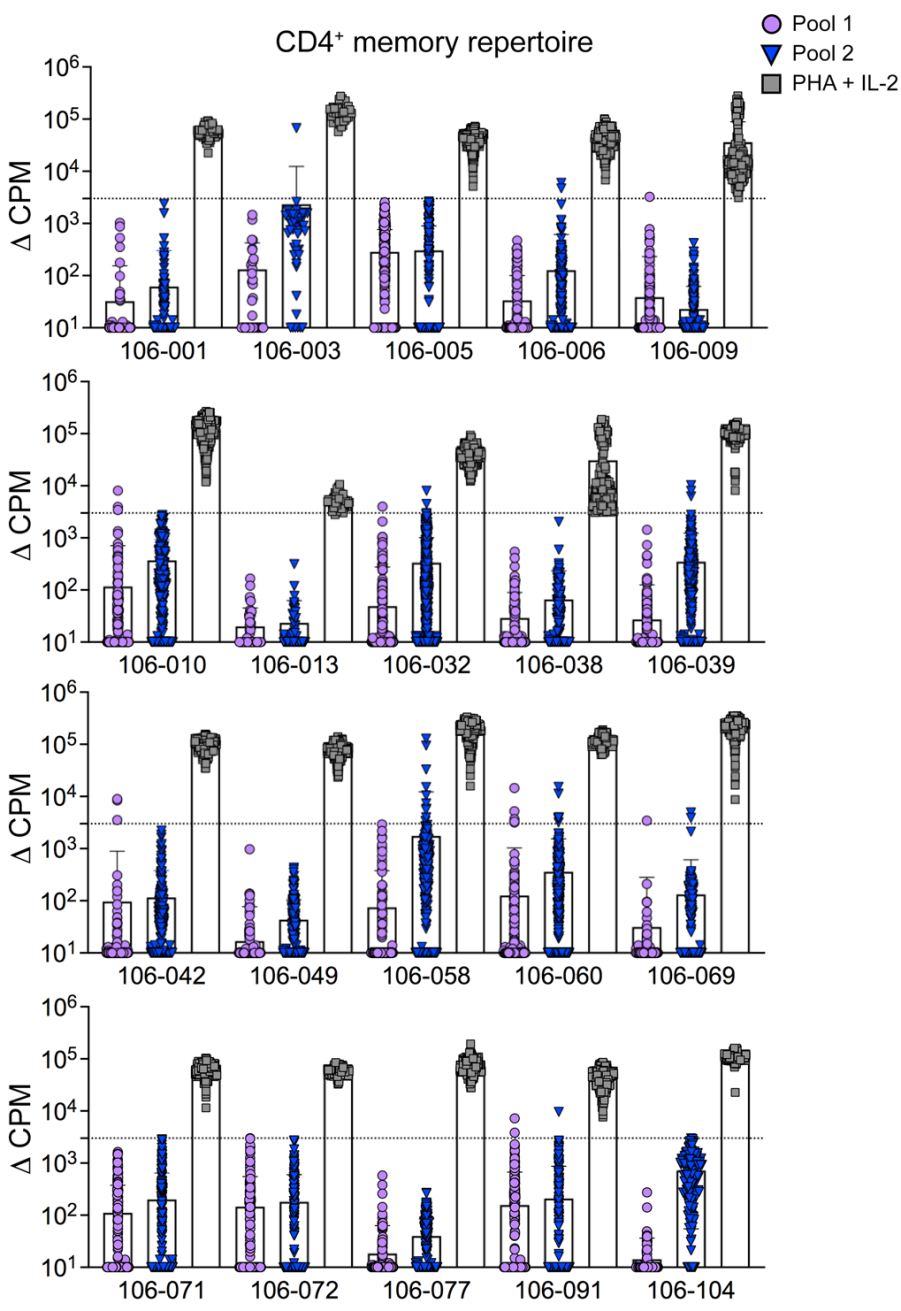
The preimmunization repertoire of Env-specific memory CD4^+^ T cells. Preimmunization repertoires of 20 donors were screened for Env reactivity using the T cell library method with 2 pools of overlapping peptides collectively spanning the entire consensus sequence protein (ConS). Positive control wells included phytohemagglutinin (PHA) and IL-2. Proliferative responses are shown for memory CD4^+^ T cells isolated from all 20 volunteers before vaccination (V2). Data are shown after background subtraction (mean ± SD). Positive responses were defined as greater than 3,000 cpm, with a stimulation index greater than 5 (dotted line).

CD4^+^ T cell responses to antigens are rarely detected in standard IFN-γ enzyme-linked immunospot (ELISpot) assays performed with peripheral blood mononuclear cells (PBMCs) from nonimmune blood donors prior to vaccination or infection (18–20). We therefore used a sensitive T cell library method (8, 21) to examine the gp160 Env reactivity of naive (CD45RA^+^CCR7^+^) and memory (CD45RA^−^CCR7^−/+^) CD4^+^ T cells isolated by FACS from PBMCs before immunization (visit 2, V2; Supplemental Figure 1A). Naive and memory CD4^+^ T cells were seeded at limiting dilution and expanded polyclonally for 27 days. Approximately 10,000-fold expansion was achieved without clonal distortion, as shown by TCR V–specific monoclonal antibody staining (Supplemental Figure 1, B and C). An aliquot of each cell line was then tested for proliferative responses to 2 pools of overlapping peptides spanning the entire ConS Env gp160 (Supplemental Table 2) presented by irradiated autologous monocytes (Figure 1). In the naive CD4^+^ repertoire, 4 volunteers exhibited responses to peptide pool 1 and 5 to peptide pool 2 (Supplemental Figure 2). In the memory CD4^+^ repertoire, 7 donors exhibited responses to peptide pool 1 and 8 to peptide pool 2 (Figure 1). Memory responses to both peptide pools were detected in 4 donors. The frequencies of responses detected in memory and naive cell compartments were similar. Although antigen-stimulated memory T cells would be present at relatively high frequencies, those that cross react with HIV would comprise only a very small minority of the T cells responding to a non-HIV immunogen.

**Figure 2:**
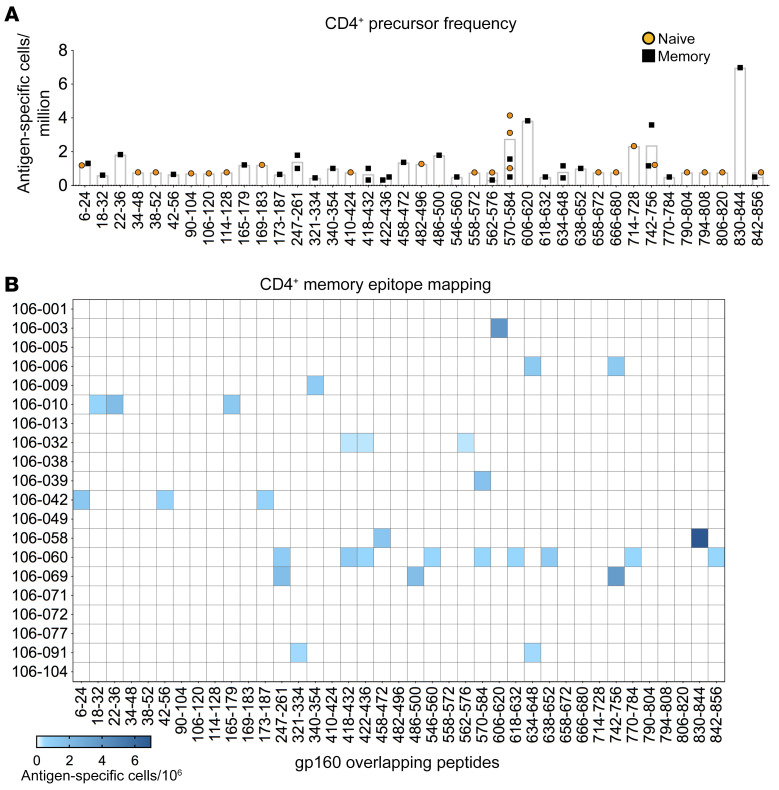
Precursor frequency and epitope specificity of preimmunization Env-reactive naive and memory CD4^+^ T cells. Env-reactive CD4^+^ T cell lines derived from the preimmunization naive and memory repertoires of 20 donors were mapped for epitope specificity. Precursor frequencies were calculated from the initial limiting dilution. (**A**) Naive and memory precursor frequencies for each specificity. Each symbol represents one CD4^+^ T cell line. Bars show mean values. (**B**) Epitope specificities and precursor frequencies determined for memory CD4^+^ T cells.

To identify the targeted epitopes, T cell reactivity was mapped using a matrix of overlapping peptides from the appropriate pool. Inferred target peptides were then tested individually to confirm the specificity of each response. The limiting dilution employed during the T cell library experiments enabled calculation of epitope response precursor frequencies in preimmune naive (Supplemental Figure 2B) and memory compartments (Figure 2). Proliferative peptide-specific responses were detected in 14 volunteers, consistent with previous estimates (8), and 39 different peptides were recognized across the entire sequence of gp160 Env. Eight peptides were recognized by more than one donor (amino acids 6–24, 247–261, 418–432, 422–436, 562–576, 570–584, 634–648, and 742–756), and 3 peptides were detected concurrently in the naive and memory repertoires of individual donors (amino acids 570–584, 742–756, and 842–856) (Figure 2B and Supplemental Figure 2B).

As these experiments were conducted at a preimmunization time point, the Env-reactive CD4^+^ T cells detected in the memory repertoires of 11 donors were most likely primed by cross-reactive antigens. Previously, we found that 83% of HIV-1 peptides recognized by the CD4^+^ memory population in HIV-1–unexposed donors had 8- to 12-amino-acid-long matches to human microbiome proteins, suggesting these may constitute one source of the antigens eliciting these CD4^+^ T cell responses (8), similar to that described by Su et al. (10).

### Postvaccination T cell responses.

**Figure 3:**
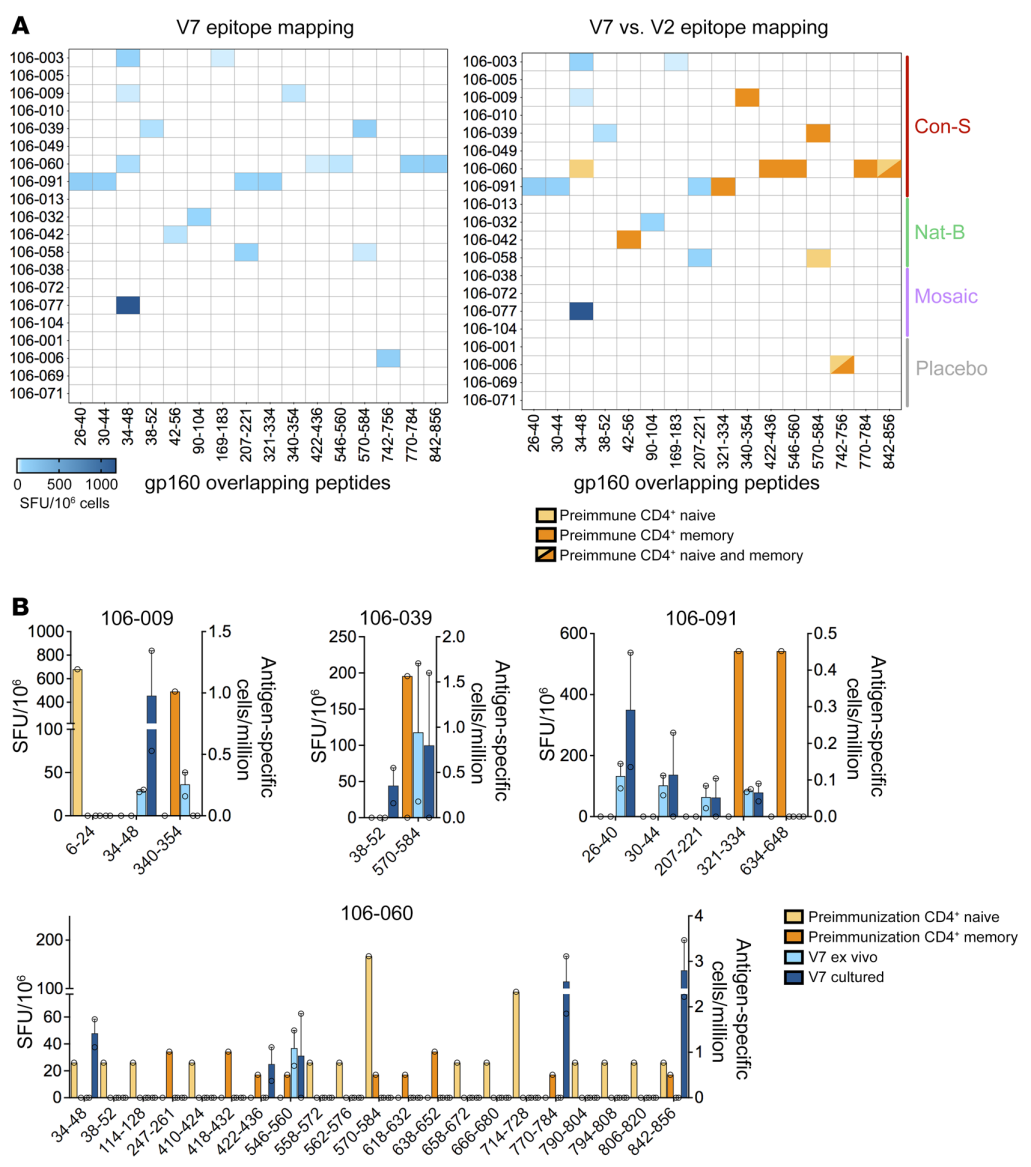
Epitope specificity of preimmunization and postvaccination Env-reactive CD4^+^ T cells. Postvaccination CD4^+^ T cell responses were mapped and quantified at V7 using ex vivo and cultured IFN-γ ELISpot assays. (**A**) Heatmaps showing the combined epitope mapping data from all volunteers at V7 (left) alongside a comparison with the epitope mapping data from all 20 volunteers at V2 (right). Ex vivo results are shown if both ex vivo and cultured data were available. Mean values are shown. SFU, spot-forming unit. (**B**) Identification of matching epitope-specific responses in the preimmunization and postvaccination repertoires of 4 donors immunized with ConS DNA. The scale for ex vivo and cultured data is shown on the left *y* axis, and the scale for naive (yellow) or memory (orange) precursor frequencies is shown on the right *y* axis. Mean ± SEM.

T cell responses to the ConS peptides were analyzed 14 days after the third DNA vaccination (visit 7, V7) and 201 days after the second MVA vaccination (visit 15, V15). Because of the limiting size of the volunteer blood samples after vaccination, the T cell library method could not be used. Responses were measured initially using an ex vivo IFN-γ ELISpot assay. PBMCs were then tested in a cultured IFN-γ ELISpot assay, after incubation with ConS peptides plus IL-2. This approach either confirmed ex vivo responses or identified subdominant responses undetectable ex vivo (18). Each assay was confirmed at least twice by different operators (Figure 3).

Similar to previous DNA immunization studies (18, 22–24), gp160 Env peptide–specific responses at V7 were detected in only 50% of donors (Figure 3A). Unblinding revealed that 5 had received ConS DNA, 3 NatB DNA, 1 Mosaic DNA, and 1 was a placebo control. Formal comparisons between responses elicited in each study arm cannot be made because of low volunteer numbers in each group, and because immune reactivity evaluation used only ConS peptides. The V7 responses, measured as IFN-γ–producing cells per 1 × 10^6^ PBMCs, were then compared with the corresponding preimmunization responses (V2), measured as proliferating epitope-specific cells per 1 × 10^6^ naive or memory CD4^+^ T cells (Figure 3A). Sampling depth in the preimmunization evaluations was limited by the number of reactive naive and memory CD4^+^ T cells. Of the 20 peptide-specific responses observed across 9 Env-immunized volunteers at V7, 10 were also detected in the corresponding preimmunization repertoires at V2, with between 1 and 5 matching the preimmunization repertoires in 4 ConS-vaccinated and 2 NatB-vaccinated donors (Figure 3A). Three responses matched the preimmunization naive CD4^+^ T cell repertoire, and 8 matched the preimmunization memory CD4^+^ T cell repertoire. One response was observed in both naive and memory CD4^+^ T cell repertoires. Postvaccination responses not detected in the corresponding preimmunization repertoires may have originated from precursors falling below the limit of detection. Notably, the placebo control volunteer (number 106-006) also exhibited a response to the same peptide at the preimmunization time point, and CD4^+^ T cells specific for this peptide were detected in the preexisting naive and memory repertoires (Figure 3A). Similar rare findings have been seen previously (18–20).

These results indicated that at least 40% (8/20) of the responses detected at V7 originated from memory CD4^+^ T cells in the preimmune repertoire. In the ConS-vaccinated group, preexisting memory CD4^+^ T cells contributed to the postvaccination CD4^+^ T cell response in 4 of 5 donors, as highlighted by a comparison of response magnitudes at V7 with V2 precursor frequencies (Figure 3B). Exact peptide matching could explain this high frequency. However, the library assay detects proliferative T cell responses in preimmunized donors and is more sensitive than the single-function IFN-γ ELISpot assay. Indeed, only 3 out of 48 responses detected in the library assays were also detected in prevaccination ex vivo IFN-γ ELISpot assays (Supplemental Table 3). Similar differences in sensitivity have been reported for preexposure T cell responses to SARS-CoV-2 (25). It is also likely that only a fraction of the naive or memory CD4^+^ T cells that proliferated at the preimmunization time point subsequently matured into effectors capable of producing IFN-γ. Thus, our measurements may underestimate the degree of expansion. Only 3 postvaccination responses arose from the detectable naive repertoire (donors 106-058 and 106-060). Although we did not conduct parallel evaluations of naive and memory CD8^+^ T cells in this study, previous experience has shown that nearly all primary responses elicited by DNA vaccines occur in CD4^+^ T cells (18, 22, 24).

Several new responses were detected at V15 after boosting with MVA, and only 5 responses were maintained from the V7 time point (Supplemental Figure 3A). The 5 ConS peptides identified had a similarity of 80% to 100% with MVA-CMDR and of 93% to 100% with ConS for the NatB donors (Supplemental Figure 3B). None of the V15 responses were observed in the preimmunization repertoires (Supplemental Figure 3C). This disparity could be explained by the sequence differences between the clade E gp150 Env in the MVA-CMDR vaccine and the gp160 Env proteins encoded by the DNA vaccines (Supplemental Figure 4A). The degree of sequence matching for each peptide between the original ConS preimmunization epitopes and the relevant sequence in the MVA-CMDR used for the boost ranged between 60% and 100% (Supplemental Figure 4B). Furthermore, 7 of the preimmunization epitopes were near the C-terminus of gp41 and so were absent from the boosting MVA-CMDR gp150 antigen. These differences probably explain why these post–MVA-CMDR responses were so different.

### Clonal sharing between preimmunization and postvaccination T cells.

**Figure 4:**
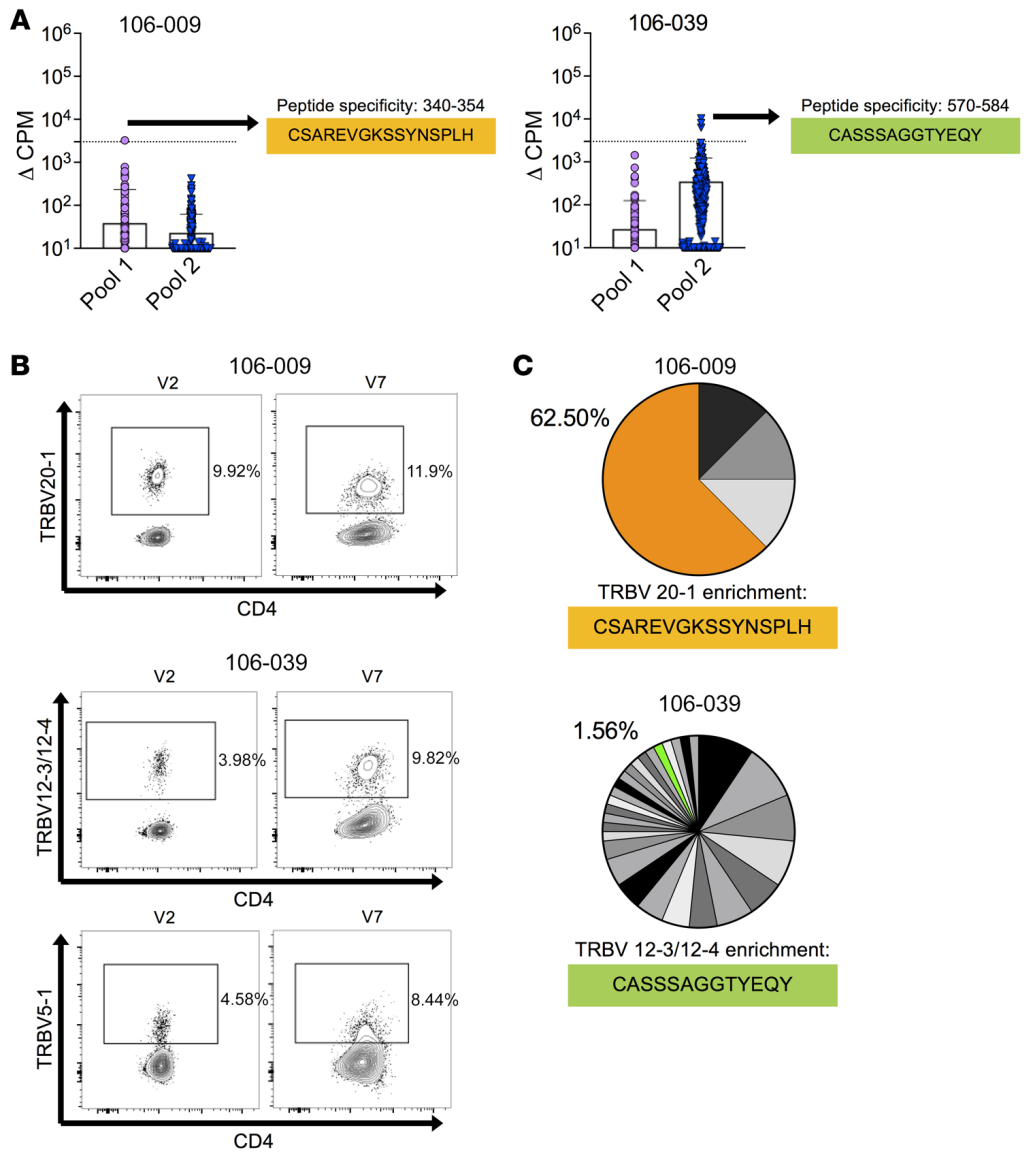
Clonotype representation in the preimmunization and postvaccination repertoires of Env-reactive CD4^+^ T cells. (**A**) CD4^+^ T cell clones were derived from preimmunization repertoires (V2) of 2 volunteers, 106-009 and 106-039, who showed matching postvaccination responses to ConS peptides (V7). Expressed *TRA* and *TRB* gene rearrangements were sequenced from mRNA. (**B**) Protein-level expression of the corresponding TCR Vβ segments at each time point determined by flow cytometry. Plots are gated on live CD3^+^ cells. (**C**) The postvaccination TCR Vβ–defined populations shown in **B** were isolated by FACS to purity. Expressed *TRB* gene rearrangements were sequenced from mRNA. Each pie chart segment represents a distinct clonotype and the matching clonotype sequences from **A**.

Env-specific clones were generated from ConS peptide–reactive memory CD4^+^ T cell lines established at the preimmunization time point, matching responses detected in the same donor at the first postvaccination time point (amino acids 340–354 in donor 106-009 and amino acids 570–584 in donor 106-039; Figure 3B). An unbiased method was used to sequence TCR mRNA. From donor 106-009, two clones, grown from a single responding line (number 180) shared an identical TRAV22/TRBV20-1^+^ TCR. From donor 106-039, seven clones from 3 responding lines (numbers 55, 62, and 99) expressed TRBV5-1, TRBV12-3, or TRBV19 (Figure 4A and Supplemental Figure 5A).

The contribution of these clonotypes to the corresponding V7 responses was assessed by FACS-isolated CD4^+^ T cells with matched Vβ TCRs. A slight increase in the frequency of TRBV20-1^+^ CD4^+^ T cells was found in donor 106-009 and small increases in the frequencies of TRBV5-1^+^ and TRBV12-3/12-4^+^ CD4^+^ T cells in donor 106-039 between V2 (before vaccination) and V7 (after serial priming with DNA; Figure 4B). These sorted populations were then sequenced at the mRNA level to characterize all expressed *TRB* gene rearrangements as markers of clonal identity. In donor 106-009, the TRBV20-1/CSAREVGKSSYNSPLH/TRBJ1-6 sequence from the original Env-specific clone was identified at a frequency of 62.5% among all TRBV20-1^+^ CD4^+^ T cells. In donor 106-039, the TRBV12-3/CASSSAGGTYEQY/TRBJ2-7 sequence from the original Env-specific clone was identified at a frequency of only 1.56% among all TRBV12-3/12-4^+^ CD4^+^ T cells (Figure 4C), whereas the clone TRBV5-1/CASSWGTGAPGGELF/TRBJ2-2 sequence was not found. Importantly, the matched sequences were identical at the nucleotide level in both donors, confirming identical clonotypes across time points. In a further experiment, cryopreserved PBMCs from V7 were cultured with the relevant Env peptides to expand the corresponding epitope-specific CD4^+^ T cells. After 10 days, memory CD4^+^ T cells were isolated by FACS and constituent clonotypes were identified by sequencing all expressed *TRB* gene rearrangements. In donor 106-009, the TRBV20-1/CSAREVGKSSYNSPLH/TRBJ1-6 sequence from the original Env-specific clone was present at a low frequency among all TRBV20-1^+^ CD4^+^ T cells, possibly because of bystander activation by IL-2 (Supplemental Figure 5, B and C).

Collectively, these experiments showed that memory clonotypes detected before immunization contributed to CD4^+^ T cell responses induced by serial priming with recombinant DNA. A limitation of this ancillary study was the small number of individuals studied. This reflected the need for leukapheresis samples to provide monocytes for the library assays, which was only possible in a subset of study participants. Nevertheless, at the level of epitope specificity, 40% of the Env-specific CD4^+^ T cell responses observed after DNA priming were derived from the preexisting memory repertoire, and the same clonotypes were found at both time points in 2 representative donors. These observations illustrate original antigenic sin in the context of a vaccine-induced primary T cell response (15, 26). However, different specificities emerged as the CD4^+^ T cell response matured over time after boosting with another Env subtype delivered by a strongly immunogenic recombinant MVA. Preexisting immunity therefore contributes to early vaccine–induced CD4^+^ T cell responses, but can be reshaped after further rounds of antigenic stimulation.

## Methods

Detailed experimental methods are included with the supplemental material.

### Study approval.

The data reported in this manuscript were generated from a substudy of the HVTN 106 trial, which was a phase I, multicenter, randomized, double-blinded trial of 3 different HIV-1 Env immunizations (clinicaltrials.gov NCT02296541). Safety monitoring was performed by the HVTN 106 Protocol Safety Review Team (PSRT) and the HVTN Safety Monitoring Board (SMB). The study was approved by the relevant Institutional Review Boards. All participants provided written informed consent prior to inclusion in accordance with the Declaration of Helsinki.

## Author contributions

SLC, EB, PB, and AJM conceived, designed, and analyzed the study. SLC, EB, and ET performed experiments. WF and BK performed additional data analysis. KL, JEM, and DAP sequenced the TCRs. NF, JMM, KWC, JRM, SRW, LRB, BFH, and BK conceived, designed, and managed the HVTN 106 vaccine trial. All authors contributed intellectually and read, edited, and approved the final manuscript. SLC and EB share first authorship. SLC is in first position because she initiated the study and conducted the T cell library experiments. EB generated CD4^+^ T cell clones, performed TCR analysis, completed the ELISpot assays, and wrote the manuscript with AJM.

## Supplementary Material

Supplemental data

## Version 1 12/01/2021

Electronic publication
