# Supplementary material for: Preexisting memory CD4+ T cells contribute to the primary response in an HIV-1 vaccine trial
Source: J Clin Invest. 2021 Dec 1;131(23):e150823. doi: 10.1172/JCI150823 (PMC8631594; doi:10.1172/JCI150823)
Supplement: Supplemental data [file jci-131-150823-s070.pdf]

SUPPLEMENTAL MATERIALS

Study design

The data reported in this manuscript were generated from a substudy of the HVTN 106 trial, which was a phase 1, multicenter, randomized, double-blinded trial of three different HIV-1 Env immunizations (clinicaltrials.gov NCT02296541). These vaccines were delivered as DNA formulations encoding the gp160 Env protein from a clade B transmitted founder virus (T/F B.1059, HV13288) (NatB), the gp160 Env protein from the group M consensus virus (HV13287) (ConS), or a trivalent gp160 Env protein (HV13284, HV13285, and HV13286) designed for optimal global coverage (Mosaic)(1). Each vaccine was administered intramuscularly at a dose of 4mg DNA on day 0 (V2), day 28 (V4), and day 56 (V6). Volunteers were then boosted with an intramuscular immunization on day 112 (V8) and day 224 (V10) with 10^8 pfu of MVA-CMDR expressing the Env gp150 protein from a clade E virus (isolate CM235) together with Gag and inactivated Pol from a clade A virus (isolate CM240). Placebo volunteers were primed and boosted with saline at the same timepoints. A total of 105 volunteers participated in the whole trial, with 30 in each Env vaccination group and 15 in the placebo group. Twenty of these volunteers provided leukapheresis samples after vaccination as a source of monocytes for the library experiments in this ancillary study.

At the time of experimentation, all those involved in this study, including the volunteers, the clinical staff, and the authors, were blinded with respect to sample identity. Samples were therefore evaluated for $CD4^+$ T cell responses to overlapping ConS peptides, which provided the best overall match across all vaccines. The study was

unblinded on completion of the full protocol, at which time it was revealed that eight volunteers had received ConS DNA, four had received NatB DNA, four had received Mosaic DNA, and four were placebo controls. The vaccination regimens are shown in **Table S1**.

The trial was conducted by the HIV Vaccine Trials Network (HVTN). The IND sponsor was the Division of AIDS (DAIDS), National Institutes of Health (NIH), Department of Health and Human Services (DHHS). The vaccines were provided by DAIDS (DNA) and the Military HIV Research Program (MHRP) at the Walter Reed Army Institute of Research (MVA).

Human samples

Venous blood samples were collected from volunteers at the HVTN clinical sites. Leukapheresis samples were collected at two of these sites (Boston and Seattle). PBMCs were isolated via standard density gradient centrifugation as described previously (2). Peripheral blood samples for the generation of allogeneic feeder cells were processed similarly from leukapheresis cones purchased from National Health Service (NHS) Blood and Transplant UK. Allogeneic feeder cells were irradiated at 45 Gy.

T cell libraries

PBMCs cryopreserved from pre-vaccination samples (V2) were thawed and enriched for $CD4^+$ T cells using a MicroBead Kit (Miltenyi Biotec). $CD14^+$ monocytes were prepared similarly from leukapheresis samples using a MicroBead Kit (Miltenyi Biotec) and cryopreserved in liquid nitrogen for use in downstream assays with autologous

$CD4^+$ T cells. $CD4^+$ T cells were stained for 15 min at room temperature with LIVE/DEAD Fixable Aqua (Thermo Fisher Scientific), anti-CD3–APC-Cy7 (clone SK7; 561800 BD Biosciences), anti-CD4–APC (clone SK3; 566915 BD Biosciences), anti-CD45RA–PacBlue (clone MEM56; MHCD45RA28 Thermo Fisher Scientific), anti-CCR7–FITC (clone 3D12; 11-1979-42 Thermo Fisher Scientific), and the dump markers anti-CD8–PE-Cy7 (clone SK1; 335787 BD Biosciences), anti-CD14–PE-Cy7 (clone M5E2; 557742 BD Biosciences), anti-CD16–PE-Cy7 (clone 3G8; 557744 BD Biosciences), anti-CD19–PE-Cy7 (clone SJ25C1; 557835 BD Biosciences), and anti-CD56–PE-Cy7 (clone B159; 557747 BD Biosciences). Live $dump^-$ $CD3^+$ $CD4^+$ T cells were sorted into naïve ($CD45RA^+$ $CCR7^+$) and memory ($CD45RA^-$ $CCR7^{-/+}$) populations (0.5 x 10^5 to 2 x 10^6 per subset) at a minimum purity of 98% using a FACSAria III (BD Biosciences).

FACS-sorted naïve and memory $CD4^+$ T cells were seeded at a limiting dilution of 3 x 10^4 cells/mL into RAB5, which comprised RPMI 1640 glutamine [-] medium supplemented with 1% non-essential amino acids, 1% sodium pyruvate, 1% glutamine, 0.1% β-mercaptoethanol, and 1% penicillin/streptomycin (all from Thermo Fisher Scientific), 5% pooled human AB sera (UK National Blood Service), and 500 U/mL IL-2 (University of Oxford). Cells were expanded with 1μg/mL PHA (Remel) in the presence of irradiated allogeneic feeder cells from four different healthy donors (0.5 x 10^6 feeder cells/mL). After 20 days, an aliquot of each line was screened for the capacity to proliferate in response to ConS. Overlapping peptides spanning the entire protein were split into two pools (pool 1 and pool 2) to avoid DMSO toxicity issues (**Table S2**). Autologous $CD14^+$ monocytes were thawed, irradiated at 45 Gy, and incubated at 3 x 10^5 cells/mL with pool 1 or pool 2 peptides (2 μg/mL) for 5 hours.

Negative control wells incorporated medium supplemented with 0.045% DMSO (Sigma-Aldrich), and positive control wells incorporated medium supplemented with 10 μg/mL PHA (Remel) and 100 U/mL IL-2 (University of Oxford). An aliquot of 2.5 x 10^6 cells/mL from each $CD4^+$ T cell line was added to the monocytes after washing and resting in fresh culture medium without IL-2 for 4 hours. After 3 days, 1 μCi/mL [^{3}H]-thymidine was added to the cultures, and proliferation was measured after a further 18 hours using a MicroBeta2 Counter (Perkin Elmer) (3-6).

Positive responses were defined as >3,000 cpm with a stimulation index >5 after background subtraction (cpm in the negative control wells), informed by previous studies of assay reproducibility (3, 4). These cell lines were cryopreserved for further analysis. Proliferative responses were confirmed in epitope mapping experiments. The precursor frequency of responding cells per million was calculated according to the Poisson distribution (3-6). The 95% confidence intervals were determined according to the modified Wald method (7).

To confirm unbiased clonal expansion, live naïve-enriched ($CD45RA^+$) and memory-enriched ($CD45RA^-$) $CD4^+$ T cells were FACS-sorted as above using a FACSAria III (BD Biosciences). Cells were then stained using a TCR Vβ Repertoire Kit (IOTest Beta Mark, PMIM3497 Beckman Coulter). The expression of different TCR Vβ families determined by flow cytometry was compared on day 0 before expansion and on day 27 after expansion as described above.

Peptide pools and mapping

ConS peptides were synthesized as 15mers overlapping by 11 amino acids (total n = 212; GenScript). Peptides were split equally split into two pools (pool 1 and pool 2) and used at a final concentration of 2 μg/mL (**Table S2**). Individual peptides recognized by T cell lines from the pre-immunization timepoint were mapped using a matrix designed from pool 1 or pool 2 as appropriate. Matrices comprised 18 pools with 18 peptides per pool and a coverage of 3 as described previously (8). Each positive cell line was stimulated as described above and assayed for IFN-γ production via ELISpot.

Env-specific responses in PBMCs from V7 and V15 were mapped using ex vivo IFN-γ ELISpot assays employing a matrix comprising 60 pools with 18 peptides per pool and a coverage of 5 spanning all 212 peptides. Pool 1 and pool 2 peptides were used separately in cultured IFN-γ ELISpot assays from the same timepoints. Specific responses identified after matrix deconvolution were confirmed in further IFN-γ ELISpot assays with single peptides.

ELISpot assay

ELISpot plates (Millipore) were coated with anti-IFN-γ (clone 1-D1K; 1 µg/mL; 3420-2A Mabtech) and blocked with R10, which comprised RPMI 1640 glutamine [-] medium supplemented with 1% non-essential amino acids, 1% sodium pyruvate, 1% glutamine, 0.1% β-mercaptoethanol, and 1% penicillin/streptomycin (all from Thermo Fisher Scientific), and 10% fetal bovine serum (Gibco). PBMCs (2 x 10^5 cells/well) or T cell lines (4 x 10^4 cells/well) were added to the blocked plates and incubated with peptides (2 μg/mL) for 18–24 hours at 37°C. After incubation, the plates were washed

eight times in Dulbecco's phosphate buffered saline supplemented with 0.05% Tween (PBS-Tween; Sigma-Aldrich) and incubated for 2–4 hours at room temperature with biotinylated anti-IFN-γ (clone 7-B6-1; 1 μg/mL; 3420-2A Mabtech) diluted in PBS containing 0.5% bovine serum albumin (Sigma-Aldrich). The plates were then washed eight times in PBS-Tween, incubated with biotin-avidin peroxidase (Vector Laboratories) for 1 hour at room temperature, washed four times in PBS-Tween and four times in PBS, and developed for 20 minutes with AEC Substrate Solution or ImmPACT AMEC Red Peroxidase Substrate Solution (both from Vector Laboratories). Spots were counted using an automated ELISpot Reader System (Autoimmun Diagnostika GmbH).

Each assay was repeated by a different operator using separate aliquots of cryopreserved PBMCs. Independent confirmation was important in this context, because DNA typically elicits only weak primary immune responses in humans (9). Positive responses were defined as >20 SFU/10^6 cells in ex vivo assays and >50 SFU/10^6 cells in cultured assays after background subtraction.

Short-term cell lines

PBMCs cryopreserved from V7 and V15 were thawed and cultured for 10 days at 1.5 x 10^6 cells/mL in RAB10 (as for RAB5 with 10% pooled human AB sera) supplemented with pool 1 or pool 2 peptides (2 μg/mL) and 25 ng/mL IL-7 (R&D Systems). Cultures were supplemented on days 3 and 7 with 1,800 U/mL IL-2 (University of Oxford) in fresh RAB10. After 10 days, cells were washed three times and rested for 30 hours in RAB10.

Peptide-specific CD4$^+$ T cell clones

Reactive lines from the T cell library were thawed and rested for 2–3 hours in RAB5. Autologous CD14$^+$ monocytes were thawed, irradiated at 45 Gy, and incubated at 3 x 10^6 cells/mL with the relevant peptides (2 μg/mL) for 3 hours. Rested CD4$^+$ T cells were added to the pulsed monocytes at a 3:1 ratio. Negative controls incorporated medium supplemented with 0.045% DMSO (Sigma-Aldrich). After 7 days, cells were stained for 15 minutes at 37°C with LIVE/DEAD Fixable Aqua (Thermo Fisher Scientific), anti-CD3–PE-Texas Red (clone 7D6; MHCD0317 Invitrogen), anti-CD4–APC-Cy7 (clone RPA-T4; 557871 BD Biosciences), anti-CD25–PE-Cy7 (clone 2A3; 335824 BD Biosciences), anti-ICOS–PE (clone C398.4A; 313508 BioLegend), and the dump marker anti-CD14–FITC (clone M5E2; 561712 BD Biosciences). Activated live T cells (CD14$^-$ CD3$^+$ CD4$^+$ CD25$^+$ ICOS$^+$) were sorted using a FACSAria III (BD Biosciences) and seeded at 0.4 cells/well into 384-well plates (Corning). Cells were expanded with 1 μg/mL PHA (Remel) in the presence of irradiated allogeneic feeder cells from three different healthy donors (0.5 x 10^6 feeder cells/mL) in RAB5 supplemented with 500 U/mL IL-2 (University of Oxford). After 12–14 days, T cell clones were identified and transferred into 96-well round-bottom plates (Corning). An aliquot from each clone (5x 10^4 cells/mL) was stimulated with the relevant peptide (2 μg/mL) after washing and resting in fresh RAB5 without IL-2 for 5 hours. IFN-γ production was measured via ELISpot. Positive clones were defined as >50 SFU/10^6 cells after background subtraction.

TCR sequencing

Peptide-specific CD4$^+$ T cell clones were stained as described above for viability and surface expression of CD3 and CD4. A maximum of 5 x 10^3 live CD3$^+$ CD4$^+$ T cells

was sorted into 100 μL of RNAlater (Ambion) using a FACSAria III (BD Biosciences). All expressed *TRA* and *TRB* gene transcripts were amplified using an unbiased template-switch anchored RT-PCR (10). Amplicons were sub-cloned, sampled, and sequenced as described previously (11). Gene use was assigned using the ImMunoGeneTics (IMGT) nomenclature (12).

SUPPLEMENTAL FIGURES AND FIGURE LEGENDS

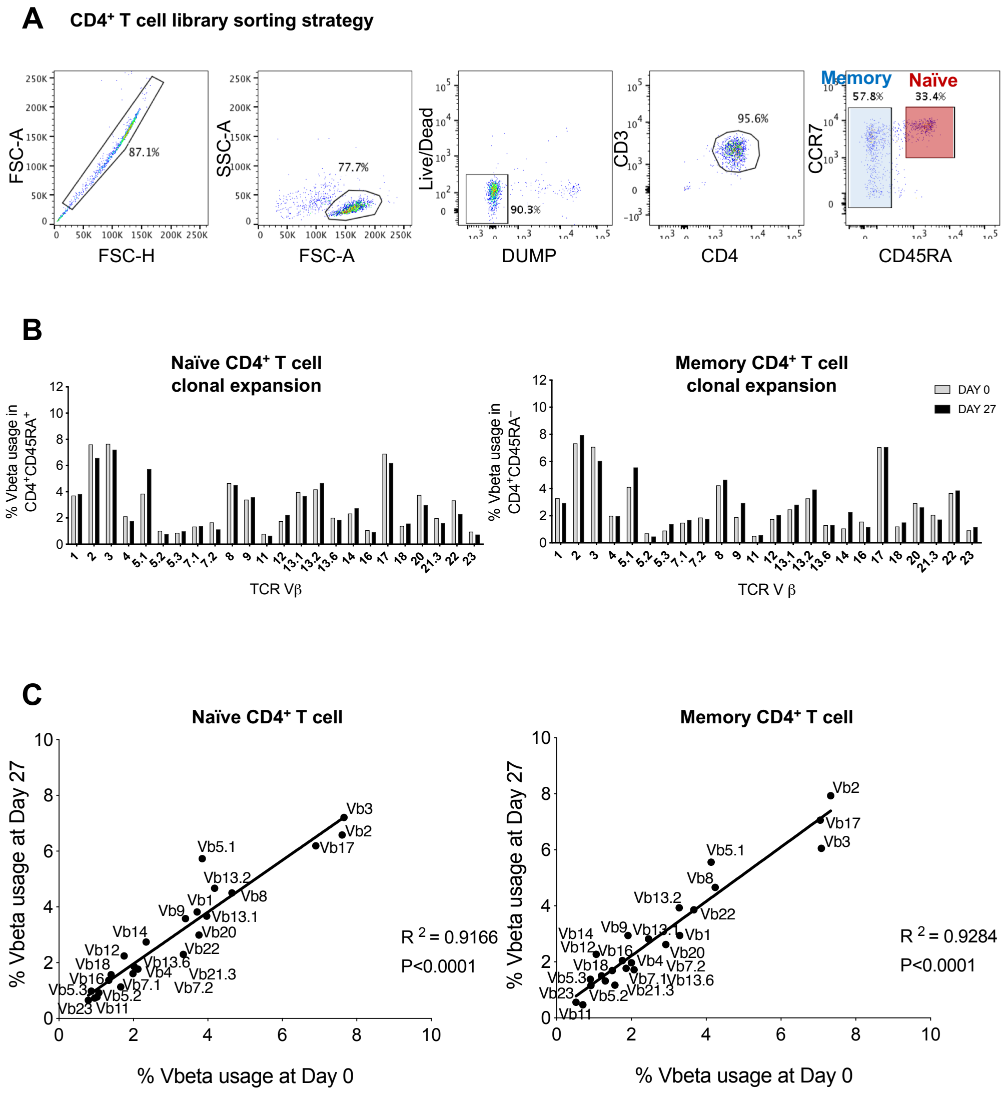

Figure S1. Isolation and expansion of pre-immunization naïve and memory CD4⁺ T cells. (**A**) Flow cytometric gating strategy for the purification of naïve and memory CD4⁺ T cells. Cells were gated serially as singlets, lymphocytes, and Aqua⁻ CD8⁻ CD14⁻ CD16⁻ CD19⁻ CD56⁻ events, and then as naïve (CD45RA⁺ CCR7⁺) or memory (CD45RA⁻ CCR7⁻/⁺) CD3⁺ CD4⁺ T cells. (**B**) Naïve and memory CD4⁺ T cells were tested for protein-level expression of the indicated TCR Vβ segments before (day 0) and after expansion (day 27). Data are shown from one representative donor. (**C**) Correlations between percent expression of each TCR Vβ segment at day 0 and day 27. Significance was assessed using the Spearman rank test.

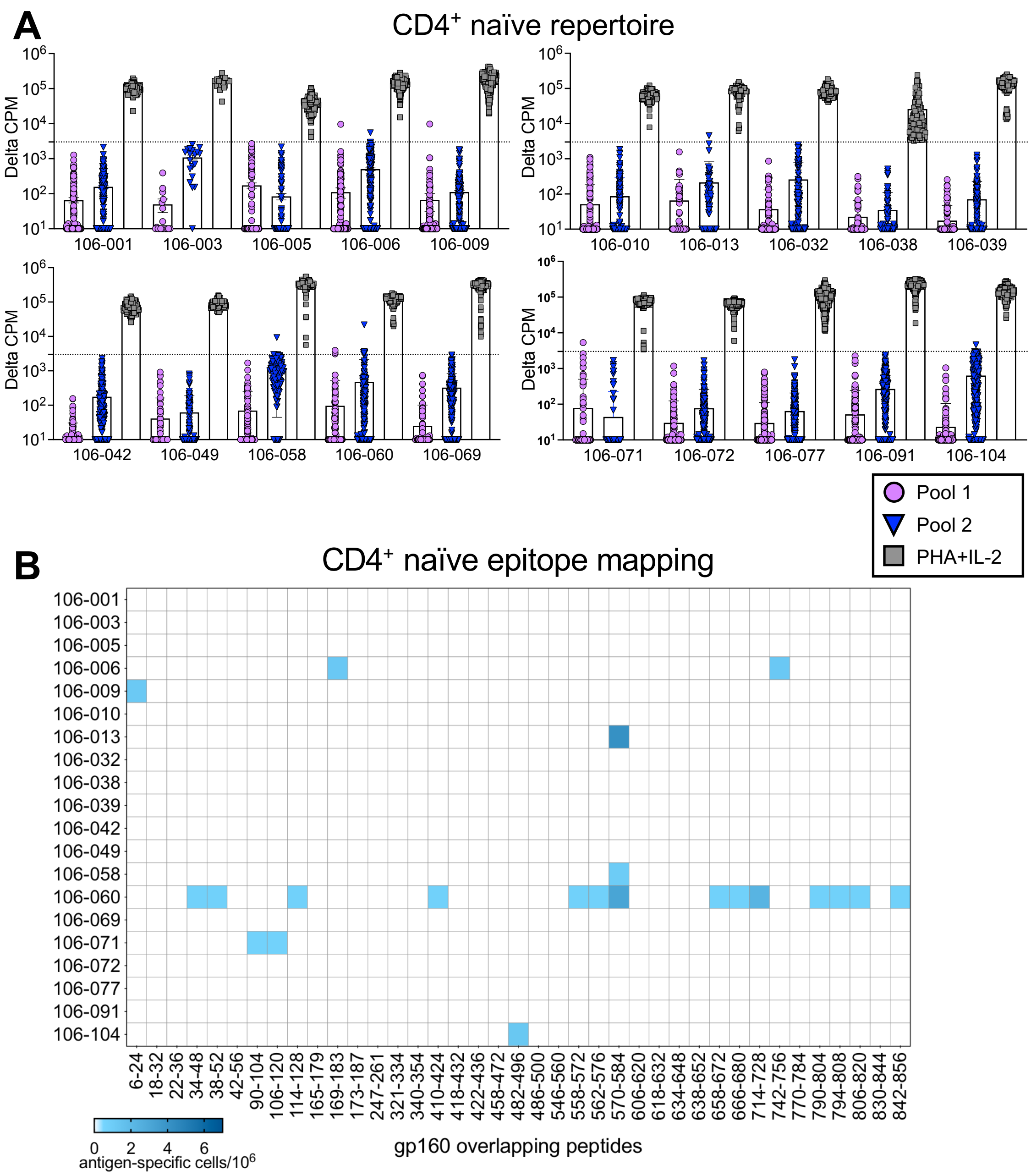

Figure S2. The pre-immunization repertoire and epitope specificity of Env-specific naïve CD4⁺ T cells. Pre-immunization repertoires of 20 donors were screened for Env reactivity using the T cell library method with two pools of overlapping peptides collectively spanning the entire consensus sequence protein (ConS). Positive control wells included phytohemagglutinin (PHA) and IL-2. Proliferative responses are shown for naïve (**A**) before vaccination (V2). Data are shown after background subtraction (mean ± SD). Positive responses were defined as >3,000 cpm with a stimulation index >5 (dotted line). Env-reactive CD4⁺ T cell lines derived from the pre-immunization naïve were mapped for epitope specificity and the precursor frequency was calculated (**B**).

A

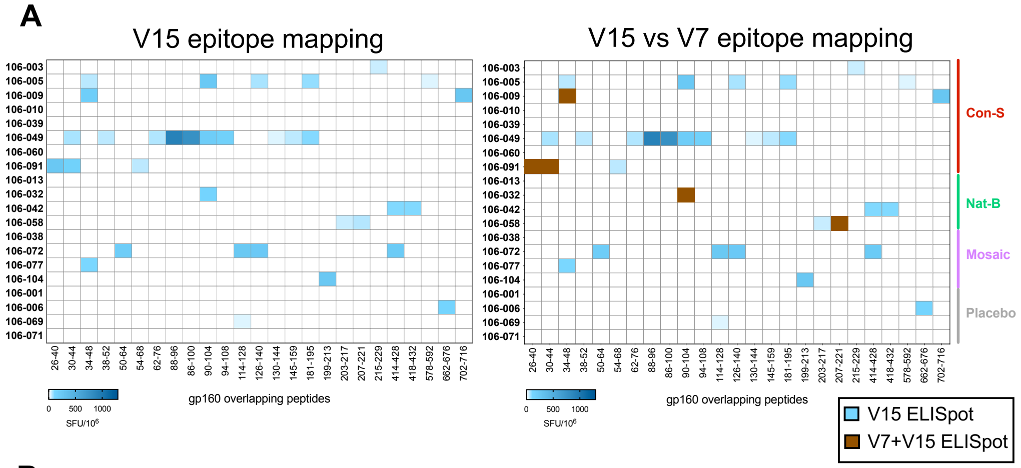

B

PEPTIDE ID	PEPTIDE SEQUENCE	Identity with MVA-CMDR	Identity with NatB
26-40	MICSAAENLWVTVYY	80%	(not from NatB donor)
30-44	AAENLWVTVYYGVPV	87%	(not from NatB donor)
34-48	LWVTVYYGVPVWKEA	87%	(not from NatB donor)
90-104	TENFNMWKNNMVEQM	100%	100%
207-221	KVSFEPIPIHYCAPA	80%	93%

C

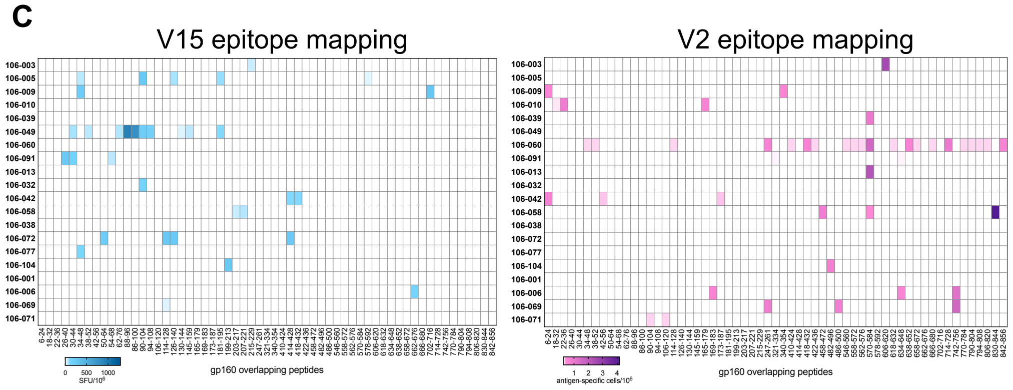

Figure S3. Epitope specificity of Env-reactive CD4$^+$ T cells after boosting with MVA-CMDR. Post-vaccination CD4$^+$ T cell responses were mapped and quantified at V15 using ex vivo and cultured IFN-γ ELISpot assays. (**A**) Heatmaps showing the combined epitope mapping data from all volunteers at V15 (left) alongside a comparison with the epitope mapping data from all volunteers at V7 (right). Ex vivo results are shown if both ex vivo and cultured data were available. (**B**) Sequence similarity (% identity of amino acids, determined by alignment of peptide sequences with the MVA-CMDR Env gp-150 sequence) between the five peptides from ConS-Env identified in both V7 and V15 and the MVA-CMDR sequence. Peptides from ConS-Env identified in NatB donors have also been aligned to the NatB Env sequence. (**C**) Heatmaps showing a comparison between response magnitudes from all volunteers at V15 (left) and precursor frequencies from all volunteers at V2 (right). Data are shown as mean values. SFU, spot-forming unit.

A

Whole sequence comparison	Overall Identity %
ConS vs NatB	80%
ConS vs Mosaic1	85%
ConS vs Mosaic2	84%
ConS vs Mosaic3	82%
ConS vs MVA-CMDR	80%
NatB vs MVA-CMDR	74%
Mosaic1 vs MVA-CMDR	75%
Mosaic2 vs MVA-CMDR	77%
Mosaic3 vs MVA-CMDR	75%

B

PEPTIDE ID	PEPTIDE SEQUENCE	Single Identity with MVA-CMDR
6-24	NCQHLWRWGTLILGM	75%
18-32	GTLILGMLMICSAAE	67%
22-36	LGMLMICSAAENLWV	67%
34-48	LWVTVYYGVPVWKEA	87%
38-52	VYYGVPVWKEANTTL	80%
42-56	VPVWKEANTTLFCAS	80%
90-104	TENFNMWKNNMVEQM	100%
106-120	EDIISLWDQSLKPCV	93%
114-128	QSLKPCVKLTPLCVT	100%
165-179	IRDKKQKVYALFYRL	80%
169-183	KQKVYALFYRLDVVP	80%
173-187	YALFYRLDVVPIDDN	73%
247-261	CTHGIKPVVSTQLLL	100%
321-334	GDIIGDIRQAHCNIS	73%
340-354	KTLQQVAKKLREHFN	60%
410-424	TITLPCRIKQIINMW	87%
418-432	CRIKQIINMWQGVGQ	87%
422-436	QIINMWQGVGQAMYA	93%
458-472	GNNNTNETEIFRPGG	77%
482-496	ELYKYKVVKIEPLGV	87%
486-500	YKVVKIEPLGVAPTK	80%
546-560	SGIVQQQSNLLRAIE	100%
558-572	AIEAQQHLLQLTVWG	100%
562-576	QQHLLQLTVWGIKQL	100%
570-584	VWGIKQLQARVLAVE	100%
606-620	TTVPWNSSWSNKSQD	67%
618-632	SQDEIWDNMTWMEWE	73%
634-648	EINNYTDIIYSLIEE	53%
638-652	YTDIIYSLIEESQNQ	60%
658-672	QELLALDKWASLWNW	80%
666-680	WASLWNWFDITNWLW	93%
714-728	PLSFQTLIPNPRGPD	60%
742-756	RDRSIRLVNGFLALA	not found
770-784	RLRDFILIAARTVEL	not found
790-804	WEALKYLWNLLQYWG	not found
794-808	KYLWNLLQYWGQELK	not found
806-820	ELKNSAISLLDTTAI	not found
830-844	IEVVQRACRAILNIP	not found
842-856	NIPRRIRQGLERALL	not found

Figure S4. Sequence comparison with MVA-CMDR.

(**A**) Sequence similarities (expressed as % identity in amino acids, determined from sequence alignments) between the antigens used in different arms of the study (ConS, NatB and trivalent Mosaic) and MVA-CMDR. (**B**) Sequence matching with MVA-CMDR (% identity of amino acids, determined by alignment of peptides sequences with the MVA-CMDR Env gp150 sequence) of the individual ConS peptides found to be recognised at the pre-vaccination time point.

A

Donor 106-009

Clone specific for peptide 340-354: KTLQQVAKKLREHFN

CELL LINE	CLONE ID	TRAV	TRAV CDR3	TRAJ	TRBV	TRBV CDR3	TRBJ
memory#180	66	22	CAVWSGYSTLTF	11	20-1	CSAREVGKSSYNSPLH	1-6
memory#180	23	22	CAVWSGYSTLTF	11	20-1	CSAREVGKSSYNSPLH	1-6

Donor 106-039

Clone specific for peptide 570-584: VWGIKQLQARVLAVE

CELL LINE	CLONE ID	TRAV	TRAV CDR3	TRAJ	TRBV	TRBV CDR3	TRBJ
memory#55	3	21	CAVSNTNAGKST	27	5-1	CASSWGTGAPGGELF	2-2
memory#55	3	23/DV6	CAALETGNYGQNFV	26	5-1	CASSWGTGAPGGELF	2-2
memory#55	7	21	CAVSNTNAGKST	27	5-1	CASSWGTGAPGGELF	2-2
memory#55	7	23/DV6	CAALETGNYGQNFV	26	5-1	CASSWGTGAPGGELF	2-2
memory#62	25	*n.d.*	*n.d.*	*n.d.*	12-3	CASSSAGGTYEQY	2-7
memory#99	2	*n.d.*	*n.d.*	*n.d.*	12-3	CASSSAGGTYEQY	2-7
memory#99	3	*n.d.*	*n.d.*	*n.d.*	19	CASSLAFNQPQH	1-5

B

DONOR ID	PEPTIDE-ENRICHMENT	TRBV	TRBV CDR3	TRBJ	Freq (%)
106-009	340-354	5-6	CASSSGQDRFLWY	2-7	27.78
106-009	340-354	7-2	CAESGRSNTGELF	2-2	19.44
106-009	340-354	3-1	CASSQEVLNSAFTGELF	2-2	8.33
106-009	340-354	20-1	CSASPSGRKSSYNEQF	2-1	8.33
106-009	340-354	7-2	CASSSSGTLQETQY	2-5	8.33
106-009	340-354	9	CASSVGAGTSPLH	1-6	5.56
106-009	340-354	20-1	CSARDPQGDTQY	2-3	5.56
106-009	340-354	20-1	CSAREVGKSSYNSPLH	1-6	2.78
106-009	340-354	5-1	CASSRGRGQGANEKLF	1-4	2.78
106-009	340-354	18	CASSPLSRVNNQPQH	1-5	2.78
106-009	340-354	11-3	CASSLGETYEQY	2-7	2.78
106-009	340-354	5-6	CASSLVDNTEAF	1-1	2.78
106-009	340-354	20-1	CSATYNEQF	2-1	2.78
106-039	570-584	7-2	CASSFGTGNQETQY	2-5	62.79
106-039	570-584	12-4	CASRTGTSGRYEQF	2-1	6.98
106-039	570-584	27	CASSHQGRDGYT	1-2	4.65
106-039	570-584	20-1	CSARRTAGTEAF	1-1	4.65
106-039	570-584	20-1	CSAGQVNYGYT	1-2	4.65
106-039	570-584	4-2	CASSQVWAVSTNNEQF	2-1	2.33
106-039	570-584	16	CASSPRTGLDYGYT	1-2	2.33
106-039	570-584	12-4	CASGRIGQGNNEQF	2-1	2.33
106-039	570-584	5-1	CASSLANQPGDTQY	2-3	2.33
106-039	570-584	27	CASSRTIESYEQF	2-1	2.33
106-039	570-584	19	CASSIAGNQPQH	1-5	2.33
106-039	570-584	19	CASSIAPDDGYT	1-2	2.33

C

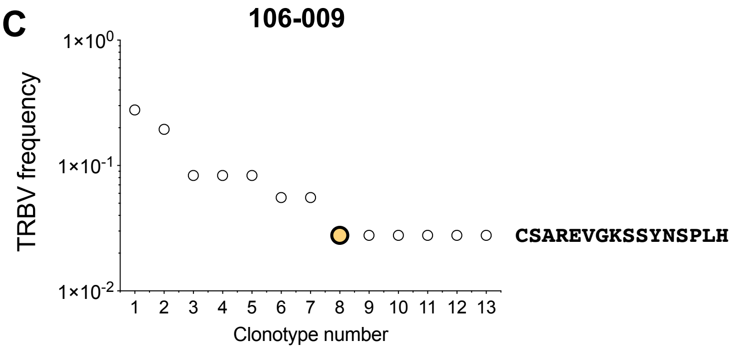

Figure S5. **Clonotype representation in the pre- and post-vaccination repertoire of Env-reactive CD4$^+$ T cells.** (**A**) CD4$^+$ T cell clones were derived from the pre-immunization repertoires (V2) of two volunteers, 106-009 and 106-039, who showed matching post-vaccination responses to ConS peptides (V7). Expressed *TRA* and *TRB* gene rearrangements were sequenced from mRNA.Cryopreserved PBMCs from V7 were cultured with the relevant Env peptides to expand the corresponding epitope-specific CD4$^+$ T cells. After 10 days, memory CD4$^+$ T cells were FACS-sorted to purity, and constituent clonotypes were identified by sequencing all expressed *TRB* gene rearrangements from mRNA. (**B**) Clonotype frequencies in donors 106-009 and 106-039. The highlighted sequence was found in the pre-immunization repertoire of donor 106-009. (**C**) Graphical representation of the data shown in A for donor 106-009.

Vaccination schedule

TIME COURSE															
VISIT	1	2	3	4	5	6	7	8	9	10	11	12	13	14	15
DAY		0	14	28	42	56	70	112	126	224	231	238	334	417	425
WEEK		0	2	4	6	8	10	16	18	32	33	34	44	55	56
**ARM**															
**Nat-B**<br>013*, 032, 042, 058		**DNA NatB**		**DNA NatB**		**DNA NatB**		MVA CMDR		MVA CMDR					
**Con-S**<br>003, 005, 009, 010, 039, 049, 060, 091		**DNA ConS**		**DNA ConS**		**DNA ConS**		MVA CMDR		MVA CMDR					
**Mosaic**<br>038, 072, 077, 104		**DNA Mosaic**		**DNA Mosaic**		**DNA Mosaic**		MVA CMDR		MVA CMDR					
Placebo<br>001, 006, 069, 071		saline		saline		saline		saline		saline					
		**Blood draw**					**Blood draw**								**Blood draw**

*volunteers are depicted in anonymized format by abbreviated publication-IP, lacking the 106 prefix.

Table S1. Schematic representation of the vaccination schedule.

A

PEPTIDE ID	SEQUENCE	PEPTIDE POOL	DESCRIPTION	POLYPROTEIN
1-16	MRVRGIQRNCQHLWR	POOL 1	HVTN106 Con S	gp160
2-20	GIQRNCQHLWRWGTL	POOL 1	HVTN106 Con S	gp160
6-24	NCQHLWRWGTLILGM	POOL 1	HVTN106 Con S	gp160
14-28	LWRWGTLILGMLMIC	POOL 1	HVTN106 Con S	gp160
18-32	GTLILGMLMICSAAE	POOL 1	HVTN106 Con S	gp160
22-36	LGMLMICSAAENLWV	POOL 1	HVTN106 Con S	gp160
26-40	MICSAAENLWVTVYY	POOL 1	HVTN106 Con S	gp160
30-44	AAENLWVTVYYGVPV	POOL 1	HVTN106 Con S	gp160
34-48	LWVTVYYGVPVWKEA	POOL 1	HVTN106 Con S	gp160
38-52	VYYGVPVWKEANTTL	POOL 1	HVTN106 Con S	gp160
42-56	VPVWKEANTTLFCAS	POOL 1	HVTN106 Con S	gp160
46-60	KEANTTLFCASDAKA	POOL 1	HVTN106 Con S	gp160
50-64	TTLFCASDAKAYDTE	POOL 1	HVTN106 Con S	gp160
54-68	CASDAKAYDTEVHNV	POOL 1	HVTN106 Con S	gp160
58-72	AKAYDTEVHNVWATH	POOL 1	HVTN106 Con S	gp160
62-76	DTEVHNVWATHACVP	POOL 1	HVTN106 Con S	gp160
66-80	HNVWATHACVPTDPN	POOL 1	HVTN106 Con S	gp160
70-84	ATHACVPTDPNPQEI	POOL 1	HVTN106 Con S	gp160
74-88	CVPTDPNPQEIVLEN	POOL 1	HVTN106 Con S	gp160
78-92	DPNPQEIVLENVTEN	POOL 1	HVTN106 Con S	gp160
82-90	QEIVLENVT	POOL 1	HVTN106 Con S	gp160
88-96	NVTENFNMW	POOL 1	HVTN106 Con S	gp160
86-100	LENVTENFNMWKNNM	POOL 1	HVTN106 Con S	gp160
90-104	TENFNMWKNNMVEQM	POOL 1	HVTN106 Con S	gp160
94-108	NMWKNNMVEQMHEDI	POOL 1	HVTN106 Con S	gp160
98-112	NNMVEQMHEDIISLW	POOL 1	HVTN106 Con S	gp160
102-116	EQMHEDIISLWDQSL	POOL 1	HVTN106 Con S	gp160
106-120	EDIISLWDQSLKPCV	POOL 1	HVTN106 Con S	gp160
110-125	SLWDQSLKPCVKLTP	POOL 1	HVTN106 Con S	gp160
114-128	QSLKPCVKLTPLCVT	POOL 1	HVTN106 Con S	gp160
118-132	PCVKLTPLCVTLNCT	POOL 1	HVTN106 Con S	gp160
122-136	LTPLCVTLNCTNVNV	POOL 1	HVTN106 Con S	gp160
126-140	CVTLNCTNVNVTNTT	POOL 1	HVTN106 Con S	gp160
130-144	NCTNVNVTNTTNNTE	POOL 1	HVTN106 Con S	gp160
140-153	VNVTNTTNNTEEKGE	POOL 1	HVTN106 Con S	gp160
141-155	NTTNNTEEKGEIKNC	POOL 1	HVTN106 Con S	gp160
145-159	TNNTEEKGEIKNCSF	POOL 1	HVTN106 Con S	gp160
149-163	EEKGEIKNCSFNITT	POOL 1	HVTN106 Con S	gp160
153-167	EIKNCSFNITTEIRD	POOL 1	HVTN106 Con S	gp160
157-171	CSFNITTEIRDKKQK	POOL 1	HVTN106 Con S	gp160
161-175	ITTEIRDKKQKVYAL	POOL 1	HVTN106 Con S	gp160
165-179	IRDKKQKVYALFYRL	POOL 1	HVTN106 Con S	gp160
169-183	KQKVYALFYRLDVVP	POOL 1	HVTN106 Con S	gp160
173-187	YALFYRLDVVPIDDN	POOL 1	HVTN106 Con S	gp160
177-190	YRLDVVPIDDNNNNS	POOL 1	HVTN106 Con S	gp160
181-195	VVPIDDNNNNSSNYR	POOL 1	HVTN106 Con S	gp160
184-197	DDNNNNSSNYRLINC	POOL 1	HVTN106 Con S	gp160
185-197	NNSSNYRLINCNTSA	POOL 1	HVTN106 Con S	gp160
188-202	NYRLINCNTSAITQA	POOL 1	HVTN106 Con S	gp160
191-205	YRLINCNTSAITQAC	POOL 1	HVTN106 Con S	gp160
195-209	NCNTSAITQACPKVS	POOL 1	HVTN106 Con S	gp160
199-213	SAITQACPKVSFEPI	POOL 1	HVTN106 Con S	gp160
203-217	QACPKVSFEPIPIHY	POOL 1	HVTN106 Con S	gp160
207-221	KVSFEPIPIHYCAPA	POOL 1	HVTN106 Con S	gp160
211-225	EPIPIHYCAPAGFAI	POOL 1	HVTN106 Con S	gp160
215-229	IHYCAPAGFAILKCN	POOL 1	HVTN106 Con S	gp160
219-233	APAGFAILKCNDKKF	POOL 1	HVTN106 Con S	gp160
223-237	FAILKCNDKKFNGTG	POOL 1	HVTN106 Con S	gp160
227-241	KCNDKKFNGTGPCKN	POOL 1	HVTN106 Con S	gp160
231-245	KKFNGTGPCKNVSTV	POOL 1	HVTN106 Con S	gp160
235-249	GTGPCKNVSTVQCTH	POOL 1	HVTN106 Con S	gp160
239-253	CKNVSTVQCTHGIKP	POOL 1	HVTN106 Con S	gp160
243-257	STVQCTHGIKPVVST	POOL 1	HVTN106 Con S	gp160
247-261	CTHGIKPVVSTQLLL	POOL 1	HVTN106 Con S	gp160
251-265	IKPVVSTQLLLNGSL	POOL 1	HVTN106 Con S	gp160
255-269	VSTQLLLNGSLAEEE	POOL 1	HVTN106 Con S	gp160
259-273	LLLNGSLAEEEIIIR	POOL 1	HVTN106 Con S	gp160
263-277	GSLAEEEIIIRSENI	POOL 1	HVTN106 Con S	gp160
267-281	EEEIIIRSENITNNA	POOL 1	HVTN106 Con S	gp160
271-285	IIRSENITNNAKTII	POOL 1	HVTN106 Con S	gp160
275-289	ENITNNAKTIIVQLN	POOL 1	HVTN106 Con S	gp160
279-293	NNAKTIIVQLNESVE	POOL 1	HVTN106 Con S	gp160
283-297	TIIVQLNESVEINCT	POOL 1	HVTN106 Con S	gp160
287-301	QLNESVEINCTRPNN	POOL 1	HVTN106 Con S	gp160
291-305	SVEINCTRPNNNTRK	POOL 1	HVTN106 Con S	gp160
295-309	NCTRPNNNTRKSIRI	POOL 1	HVTN106 Con S	gp160
299-315	PNNNTRKSIRIGPGQ	POOL 1	HVTN106 Con S	gp160
303-319	TRKSIRIGPGQAFYA	POOL 1	HVTN106 Con S	gp160
309-323	IRIGPGQAFYATGDI	POOL 1	HVTN106 Con S	gp160
313-326	PGQAFYATGDIIGDI	POOL 1	HVTN106 Con S	gp160
317-330	FYATGDIIGDIRQAH	POOL 1	HVTN106 Con S	gp160
321-334	GDIIGDIRQAHCNIS	POOL 1	HVTN106 Con S	gp160
324-338	GDIRQAHCNISGTKW	POOL 1	HVTN106 Con S	gp160
328-342	QAHCNISGTKWNKTL	POOL 1	HVTN106 Con S	gp160
332-346	NISGTKWNKTLQQVA	POOL 1	HVTN106 Con S	gp160
336-350	TKWNKTLQQVAKKLR	POOL 1	HVTN106 Con S	gp160
340-354	KTLQQVAKKLREHFN	POOL 1	HVTN106 Con S	gp160
344-359	QVAKKLREHFNNKTI	POOL 1	HVTN106 Con S	gp160
348-363	KLREHFNNKTIIFKP	POOL 1	HVTN106 Con S	gp160
352-367	HFNNKTIIFKPSSGG	POOL 1	HVTN106 Con S	gp160
357-371	KTIIFKPSSGGDLEI	POOL 1	HVTN106 Con S	gp160
361-375	FKPSSGGDLEITTHS	POOL 1	HVTN106 Con S	gp160
365-379	SGGDLEITTHSFNCR	POOL 1	HVTN106 Con S	gp160
369-383	LEITTHSFNCRGEFF	POOL 1	HVTN106 Con S	gp160
373-387	THSFNCRGEFFYCNT	POOL 1	HVTN106 Con S	gp160
377-391	NCRGEFFYCNTSGLF	POOL 1	HVTN106 Con S	gp160
381-395	EFFYCNTSGLFNSTW	POOL 1	HVTN106 Con S	gp160
385-399	CNTSGLFNSTWIGNG	POOL 1	HVTN106 Con S	gp160
389-404	GLFNSTWIGNGTKNN	POOL 1	HVTN106 Con S	gp160
393-409	STWIGNGTKNNNNTN	POOL 1	HVTN106 Con S	gp160
396-413	GNGTKNNNNTNDTIT	POOL 1	HVTN106 Con S	gp160
403-416	KNNNNTNDTITLPCR	POOL 1	HVTN106 Con S	gp160
406-420	NTNDTITLPCRIKQI	POOL 1	HVTN106 Con S	gp160
410-424	TITLPCRIKQIINMW	POOL 1	HVTN106 Con S	gp160
414-428	ITLPCRIKQIINMWQ	POOL 1	HVTN106 Con S	gp160
418-432	CRIKQIINMWQGVGQ	POOL 1	HVTN106 Con S	gp160
422-436	QIINMWQGVGQAMYA	POOL 1	HVTN106 Con S	gp160

B

PEPTIDE ID	SEQUENCE	PEPTIDE POOL	DESCRIPTION	POLYPROTEIN
426-440	MWQGVGQAMYAPPIE	POOL 2	HVTN106 Con S	gp160
430-444	VGQAMYAPPIEGKIT	POOL 2	HVTN106 Con S	gp160
434-448	MYAPPIEGKITCKSN	POOL 2	HVTN106 Con S	gp160
438-452	PIEGKITCKSNITGL	POOL 2	HVTN106 Con S	gp160
442-456	KITCKSNITGLLLTR	POOL 2	HVTN106 Con S	gp160
446-460	KSNITGLLLTRDGGN	POOL 2	HVTN106 Con S	gp160
450-463	TGLLLTRDGGNNNTN	POOL 2	HVTN106 Con S	gp160
454-468	LTRDGGNNNTNETEIF	POOL 2	HVTN106 Con S	gp160
458-472	GNNNTNETEIFRPGG	POOL 2	HVTN106 Con S	gp160
462-476	TNETEIFRPGGGDMR	POOL 2	HVTN106 Con S	gp160
466-480	EIFRPGGGDMRDNWR	POOL 2	HVTN106 Con S	gp160
470-484	PGGGDMRDNWRSELY	POOL 2	HVTN106 Con S	gp160
474-488	DMRDNWRSELYKYKV	POOL 2	HVTN106 Con S	gp160
478-492	NWRSELYKYKVVKIE	POOL 2	HVTN106 Con S	gp160
482-496	ELYKYKVVKIEPLGV	POOL 2	HVTN106 Con S	gp160
486-500	YKVVKIEPLGVAPTK	POOL 2	HVTN106 Con S	gp160
490-504	KIEPLGVAPTKAKRR	POOL 2	HVTN106 Con S	gp160
494-508	LGVAPTKAKRRVVER	POOL 2	HVTN106 Con S	gp160
498-512	PTKAKRRVVEREKRA	POOL 2	HVTN106 Con S	gp160
502-516	KRRVVEREKRAVGIG	POOL 2	HVTN106 Con S	gp160
506-520	VEREKRAVGIGAVFL	POOL 2	HVTN106 Con S	gp160
510-524	KRAVGIGAVFLGFLG	POOL 2	HVTN106 Con S	gp160
524-528	GIGAVFLGFLGAAGS	POOL 2	HVTN106 Con S	gp160
518-532	VFLGFLGAAGSTMGA	POOL 2	HVTN106 Con S	gp160
522-536	FLGAAGSTMGAASIT	POOL 2	HVTN106 Con S	gp160
526-540	AGSTMGAASITLTVQ	POOL 2	HVTN106 Con S	gp160
530-544	MGAASITLTVQARQL	POOL 2	HVTN106 Con S	gp160
534-548	SITLTVQARQLLSGI	POOL 2	HVTN106 Con S	gp160
538-552	TVQARQLLSGIVQQQ	POOL 2	HVTN106 Con S	gp160
542-556	RQLLSGIVQQQSNLL	POOL 2	HVTN106 Con S	gp160
546-560	SGIVQQQSNLLRAIE	POOL 2	HVTN106 Con S	gp160
550-564	QQQSNLLRAIEAQQH	POOL 2	HVTN106 Con S	gp160
554-568	NLLRAIEAQQHLLQL	POOL 2	HVTN106 Con S	gp160
558-572	AIEAQQHLLQLTVWG	POOL 2	HVTN106 Con S	gp160
562-576	QQHLLQLTVWGIKQL	POOL 2	HVTN106 Con S	gp160
566-580	LQLTVWGIKQLQARV	POOL 2	HVTN106 Con S	gp160
570-584	VWGIKQLQARVLAVE	POOL 2	HVTN106 Con S	gp160
574-588	KQLQARVLAVERYLK	POOL 2	HVTN106 Con S	gp160
578-592	ARVLAVERYLKDQQL	POOL 2	HVTN106 Con S	gp160
582-596	AVERYLKDQQLLGIW	POOL 2	HVTN106 Con S	gp160
586-600	YLKDQQLLGIWGCSG	POOL 2	HVTN106 Con S	gp160
590-604	QQLLGIWGCSGKLIC	POOL 2	HVTN106 Con S	gp160
594-608	GIWGCSGKLICTTTV	POOL 2	HVTN106 Con S	gp160
598-612	CSGKLICTTTVPWNS	POOL 2	HVTN106 Con S	gp160
602-616	LICTTTVPWNSSWSN	POOL 2	HVTN106 Con S	gp160
606-620	TTVPWNSSWSNKSQD	POOL 2	HVTN106 Con S	gp160
610-624	WNSSWSNKSQDEIWD	POOL 2	HVTN106 Con S	gp160
614-628	WSNKSQDEIWDNMTW	POOL 2	HVTN106 Con S	gp160
618-632	SQDEIWDNMTWMEWE	POOL 2	HVTN106 Con S	gp160
622-636	IWDNMTWMEWEREIN	POOL 2	HVTN106 Con S	gp160
626-640	MTWMEWEREINNYTD	POOL 2	HVTN106 Con S	gp160
630-644	EWEREINNYTDIIYS	POOL 2	HVTN106 Con S	gp160
634-648	EINNYTDIIYSLIEE	POOL 2	HVTN106 Con S	gp160
638-652	YTDIIYSLIEESQNQ	POOL 2	HVTN106 Con S	gp160
642-656	IYSLIEESQNQQEKN	POOL 2	HVTN106 Con S	gp160
646-660	IEESQNQQEKNEQEL	POOL 2	HVTN106 Con S	gp160
650-664	QNQQEKNEQELLALD	POOL 2	HVTN106 Con S	gp160
654-668	EKNEQELLALDKWAS	POOL 2	HVTN106 Con S	gp160
658-672	QELLALDKWASLWNW	POOL 2	HVTN106 Con S	gp160
662-676	ALDKWASLWNWFDIT	POOL 2	HVTN106 Con S	gp160
666-680	WASLWNWFDITNWLW	POOL 2	HVTN106 Con S	gp160
670-684	WNWFDITNWLWYIKI	POOL 2	HVTN106 Con S	gp160
670-684	DITNWLWYIKIFIMI	POOL 2	HVTN106 Con S	gp160
678-692	WLWYIKIFIMIVGGL	POOL 2	HVTN106 Con S	gp160
682-696	IKIFIMIVGGLIGLR	POOL 2	HVTN106 Con S	gp160
686-700	IMIVGGLIGLRIVFA	POOL 2	HVTN106 Con S	gp160
690-704	GGLIGLRIVFAVLSI	POOL 2	HVTN106 Con S	gp160
694-708	GLRIVFAVLSIVNRV	POOL 2	HVTN106 Con S	gp160
698-712	VFAVLSIVNRVRQGY	POOL 2	HVTN106 Con S	gp160
702-716	LSIVNRVRQGYSPLS	POOL 2	HVTN106 Con S	gp160
706-720	NRVRQGYSPLSFQTL	POOL 2	HVTN106 Con S	gp160
710-724	QGYSPLSFQTLIPNP	POOL 2	HVTN106 Con S	gp160
714-728	PLSFQTLIPNPRGPD	POOL 2	HVTN106 Con S	gp160
718-732	QTLIPNPRGPDRPEG	POOL 2	HVTN106 Con S	gp160
722-736	PNPRGPDRPEGIEEE	POOL 2	HVTN106 Con S	gp160
726-740	GPDRPEGIEEEGGEQ	POOL 2	HVTN106 Con S	gp160
730-744	PEGIEEEGGEQDRDR	POOL 2	HVTN106 Con S	gp160
734-748	EEEGGEQDRDRSIRL	POOL 2	HVTN106 Con S	gp160
738-752	GEQDRDRSIRLVNGF	POOL 2	HVTN106 Con S	gp160
742-756	RDRSIRLVNGFLALA	POOL 2	HVTN106 Con S	gp160
746-760	IRLVNGFLALAWDDL	POOL 2	HVTN106 Con S	gp160
750-764	NGFLALAWDDLRSLC	POOL 2	HVTN106 Con S	gp160
754-768	ALAWDDLRSLCLFSY	POOL 2	HVTN106 Con S	gp160
758-772	DDLRSLCLFSYHRLR	POOL 2	HVTN106 Con S	gp160
762-776	SLCLFSYHRLRDFIL	POOL 2	HVTN106 Con S	gp160
66-780	FSYHRLRDFILIAAR	POOL 2	HVTN106 Con S	gp160
770-784	RLRDFILIAARTVEL	POOL 2	HVTN106 Con S	gp160
774-788a	FILIAARTVELLGRK	POOL 2	HVTN106 Con S	gp160
774-788b	AARTVELLGRKGLRR	POOL 2	HVTN106 Con S	gp160
778-792	VELLGRKGLRRGWEA	POOL 2	HVTN106 Con S	gp160
782-796	GRKGLRRGWEALKYL	POOL 2	HVTN106 Con S	gp160
786-800	LRRGWEALKYLWNLL	POOL 2	HVTN106 Con S	gp160
790-804	WEALKYLWNLLQYWG	POOL 2	HVTN106 Con S	gp160
794-808	KYLWNLLQYWGQELK	POOL 2	HVTN106 Con S	gp160
798-812	NLLQYWGQELKNSAI	POOL 2	HVTN106 Con S	gp160
802-816	YWGQELKNSAISLLD	POOL 2	HVTN106 Con S	gp160
806-820	ELKNSAISLLDTTAI	POOL 2	HVTN106 Con S	gp160
810-824	SAISLLDTTAIAVAE	POOL 2	HVTN106 Con S	gp160
814-828	LLDTTAIAVAEGTDR	POOL 2	HVTN106 Con S	gp160
818-832	TAIAVAEGTDRVIEV	POOL 2	HVTN106 Con S	gp160
822-836	VAEGTDRVIEVVQRA	POOL 2	HVTN106 Con S	gp160
826-840	TDRVIEVVQRACRAI	POOL 2	HVTN106 Con S	gp160
830-844	IEVVQRACRAILNIP	POOL 2	HVTN106 Con S	gp160
834-848	QRACRAILNIPRRIR	POOL 2	HVTN106 Con S	gp160
838-852	RAILNIPRRIRQGLE	POOL 2	HVTN106 Con S	gp160
842-856	NIPRRIRQGLERALL	POOL 2	HVTN106 Con S	gp160

Table S2. Pool 1 and pool 2 peptides from ConS gp160 Env.

PUBID	ARM	PEPTIDES	PRE-IMMUNIZATION<br>V2 T cell Library (antigen-spec cell/10^6)	PRE-IMMUNIZATION<br>V2 *ex vivo* ELISpot (SFU/10^6)	POST-VACCINATION<br>V7 *ex vivo* ELISpot (SFU/10^6)	POST-VACCINATION<br>V7 cultured ELISpot (SFU/10^6)
106-003	ConS	606-620	3.831 (memory)	<20		
106-009	ConS	6-24	1.193 (naïve)	<20		
		340-354	1.012 (memory)	<20	36.25	
106-010	ConS	18-32	0.607 (memory)	<20		
		22-36	1.828 (memory)	<20		
		165-179	1.217 (memory)	<20		
106-039	ConS	570-584	1.565 (memory)	<20		100.00
106-060	ConS	34-48	0.773 (naïve)	<20		47.92
		38-52	0.773 (naïve)	<20		
		114-128	0.773 (naïve)	<20		
		247-261	1.013 (memory)	<20		
		410-424	0.773 (naïve)	<20		
		418-432	1.013 (memory)	<20		
		422-436	0.506 (memory)	<20		25.00
		546-560	0.506 (memory)	<20	36.88	31.25
		558-572	0.773 (naïve)	<20		
		562-576	0.773 (naïve)	<20		
		570-584	3.115/0.506 (naïve/memory)	<20		
		618-632	0.506 (memory)	<20		
		638-652	1.013 (memory)	22.92		
		658-672	0.773 (naïve)	<20		
		666-680	0.773 (naïve)	<20		
		714-728	2.331 (naïve)	<20		
		770-784	0.506 (memory)	<20		114.59
		790-804	0.773 (naïve)	<20		
		794-808	0.773 (naïve)	<20		
		806-820	0.773 (naïve)	<20		
		842-856	0.773 (naïve)	<20		137.50
106-091	ConS	321-334	0.452 (memory)	<20	85.50	79.17
106-013	Nat B	570-584	4.141 (naïve)	<20		
106-032	Nat B	418-432	0.323 (memory)	<20		
		422-436	0.323 (memory)	<20		
		562-576	0.323 (memory)	<20		
106-042	Nat B	6-24	1.312 (memory)	<20		
		42-56	0.654 (memory)	<20	39.84	
		173-187	0.654 (memory)	<20		
106-058	Nat B	458-472	1.828 (memory)	<20		
		570-584	1.026 (naïve)	<20	30.00	
		830-844	6.975 (memory)	<20		
106-104	Mosaic	482-496	1.28 (naïve)	83.33		
106-006	Placebo	169-183	1.214 (naïve)	<20		
		634-648	1.163 (memory)	30.00		
		742-756	1.214 /1.163 (naïve/memory)	<20	131.53	
106-069	Placebo	247-261	1.787 (memory)	<20		
		486-500	1.787 (memory)	<20		
		742-756	3.584 (memory)	<20		
106-071	Placebo	90-104	0.72 (naïve)	<20		
		106-120	0.72 (naïve)	<20		

Table S3. Ex vivo IFN-γ ELISpot data from donors in whom pre-immunization responses were detected at V2 compared with the corresponding T cell library data at V2 and the corresponding IFN-γ ELISpot data at V7.

1. Santra, S., et al., *A centralized gene-based HIV-1 vaccine elicits broad cross-clade cellular immune responses in rhesus monkeys.* PNAS, 2008. **105**(30): p. 10489-10494.

2. Bull, M., et al., *Defining blood processing parameters for optimal detection of cryopreserved antigen-specific responses for HIV vaccine trials.* J Immunol Methods, 2007. **322**(1-2): p. 57-69.

3. Campion, S.L., et al., *Proteome-wide analysis of HIV-specific naive and memory CD4(+) T cells in unexposed blood donors.* J Exp Med, 2014. **211**(7): p. 1273-80.

4. Geiger, R., et al., *Human naive and memory CD4+ T cell repertoires specific for naturally processed antigens analyzed using libraries of amplified T cells.* J Exp Med, 2009. **206**(7): p. 1525-1534.

5. Lindestam Arlehamn, C.S., et al., *Memory T cells in latent Mycobacterium tuberculosis infection are directed against three antigenic islands and largely contained in a CXCR3+CCR6+ Th1 subset.* PLoS Pathog, 2013. **9**(1): p. e1003130.

6. Mele, F., et al., *Phenotype and specificity of T cells in primary human cytomegalovirus infection during pregnancy: IL-7Rpos long-term memory phenotype is associated with protection from vertical transmission.* PLoS One, 2017. **12**(11): p. e0187731.

7. Harrell, F.E., *Regression Modeling Strategies: With Applications to Linear Models, Logistic Regression, and Survival Analysis.*, ed. S.S.B. Media. 2013: Springer.

8. Precopio, M.L., et al., *Optimizing Peptide Matrices for Identifying T-Cell Antigens.* Cytometry A, 2008. **73A**: p. 1071-1078.

9. Goonetilleke, N., et al., *Induction of multifunctional human immunodeficiency virus type 1 (HIV-1)-specific T cells capable of proliferation in healthy subjects by using a prime-boost regimen of DNA- and modified vaccinia virus Ankara-vectored vaccines expressing HIV-1 Gag coupled to CD8+ T-cell epitopes.* J Virol, 2006. **80**(10): p. 4717-28.

10. Quigley, M.F., et al., *Unbiased molecular analysis of T cell receptor expression using template-switch anchored RT-PCR.* Curr Protoc Immunol, 2011. **10**(10.33).

11. Price, D.A., et al., *Avidity for antigen shapes clonal dominance in CD8+ T cell populations specific for persistent DNA viruses.* J Exp Med, 2005. **202**(10): p. 1349-61.

12. Lefranc, M.P., *IMGT, the international ImMunoGeneTics database.* Nucleic Acids Res, 2003. **31**(1): p. 307-10.
